# Decision Letters from a Kyrgyz Research Ethics Committee (2018–2022): An Empirical Review of Transparency, Rigor, and Communication

**DOI:** 10.1177/15562646261428333

**Published:** 2026-03-03

**Authors:** Tamara Kudaibergenova, Nityanand Jain

**Affiliations:** 1 Department of Public Health and Healthcare, 185188 I.K. Akhunbaev Kyrgyz State Medical Academy, Bishkek, Kyrgyzstan; 2 Faculty of Medicine, 475768 Riga Stradinš University, Riga, Latvia; 3 Statistics Unit, Riga Stradinš University, Riga, Latvia

**Keywords:** Research ethics committee, public health, COVID-19, turnaround time, outcomes

## Abstract

Research Ethics Committees (RECs) safeguard participants, yet little is known about how their decisions are communicated to study investigators in low- and middle-income countries (LMICs). We analysed 138 decision letters from a public-health REC in Kyrgyzstan (2018–2022) using descriptive statistics and documentary review. REC panel consisted predominantly of clinical and biomedical experts, with identifying data missing for 51% (*n* = 506/991) of panelists. Lay member participation was absent despite being mandated in SOPs. The first-review approval rate was 55.1%. Communication in decision letters tended to be prescriptive rather than advisory, with critical ethical deficiencies often not flagged. Furthermore, there was a lack of explicit citation of national or international guidance. Our observations point to opportunities to improve transparency, review quality, and stakeholder engagement through standardized templates, targeted reviewer training, and the inclusion of lay members. Strengthening these practices may enhance consistency, quality, and trust in REC decision-making in comparable post-Soviet LMICs contexts.

## Introduction

The urgency of ensuring appropriate preparedness for public health emergencies has grown significantly in the wake of the COVID-19 pandemic. This need is further intensified by a range of ongoing and emerging threats, including epidemics, climate change, conflict-related migration, and natural disasters (Khatri et al., 2023). Addressing these complex challenges requires the development of resilient and responsive public health systems, capable of adapting quickly to rapidly changing circumstances. Scientific research plays a vital role in ensuring systems-level adaptability by informing preparedness and response efforts. It supports policy decisions, guides public health interventions, and contributes to the evaluation of strategies with timely, evidence-based knowledge (Embrett et al., 2025).

However, the effectiveness of such research depends not only on scientific rigor but also on the systems' capacity for prompt and high-quality ethical oversight. Research Ethics Committees (RECs) are tasked with ensuring that scientific research adheres to the established ethical standards, particularly during emergencies when the balance between urgency and ethical safeguards becomes even more difficult to maintain. Despite growing global attention to public health preparedness, the readiness of the systems responsible for organizing and reviewing scientific research, particularly the ethical review component, remains under-explored. The COVID-19 pandemic revealed significant gaps in and challenges to the functionality of RECs, especially in low- and middle-income countries (LMICs) (Burgess et al., 2023; Moodley et al., 2021).

Studies indicate that numerous RECs were caught off guard by the demands of the pandemic and lacked the operational flexibility and resources to adapt effectively in a short period. Delays in review processes, lack of emergency protocols, and limited capacity for expedited assessments were frequently reported (Canario Guzmán et al., 2022; Salamanca-Buentello et al., 2024; Tattoli et al., 2025). Our experience and discussions with REC colleagues in the Commonwealth of Independent States (CIS) region, regrettably, seem to suggest that these challenges continued to persist even post-pandemic. Many RECs still experience resource constraints, including shortages of trained personnel, limited technological support, and inadequate institutional frameworks. These factors hinder their ability to respond effectively during future public health crises. Therefore, we argue that the current approach, focused primarily on identifying surface-level problems and barriers, needs to evolve to a systems-level approach.

There is a growing need to investigate the primary causes of these challenges and to explore sustainable and context-appropriate strategies for strengthening REC operations and preparedness. In addition, it is essential to consider the specific nature of public health research, which often differs from clinical or biomedical research in both scope and methodology. Public health research typically adopts a population-based approach and engages with entire communities rather than individual patients. This approach requires meaningful community participation throughout the research process (Yuan et al., 2021). Consequently, RECs must be equipped to assess not only the protection of individual participants, but also the broader ethical implications for communities as collective stakeholders.

This raises several important questions: Are RECs prepared to ensure the protection of community interests in public health research? Can they facilitate fair and inclusive community participation? What mechanisms do they have in place to promote transparency, ensure the quality of ethical expertise, and foster communication practices that build trust? These questions point to a broader set of capacities that RECs must develop if they are to remain effective in the context of public health emergencies. In this regard, our study aims to explore the readiness of RECs in Kyrgyzstan to provide ethical review of public health research, with a particular focus on areas such as transparency, review quality, and communication styles (Box 1).

Box 1. Key definitions used in the context of REC functioning.

***Transparency***

Refers to the extent to which REC decision letters and related documentation explicitly record the REC's working procedures in relation to a given application (Rani et al., 2024; Coleman et al., 2025). This includes providing clear information on, or reference to, relevant REC standard operating protocols (SOPs), committee composition, structure and membership, and the decision-making processes used for the application under review. We also considered documentation of key performance milestones, such as the date of application submission, date of REC decision, and date of decision communication to the applicant, as part of transparency.

***Review quality***

Refers to the degree to which REC decision letters reflect a methodologically rigorous, ethically sound, and internally consistent assessment of the submitted protocol. High-quality reviews systematically identify and document ethical and methodological deficiencies, articulate these clearly in the decision letter, and ensure that the REC's internal records align with the feedback provided to investigators. The realization of quality is predicated upon the expertise of a multidisciplinary REC membership and the adherence to standardized operating procedures (McGahan et al., 2025; Tumilty et al., 2025).

***Communication style***

Describes how clearly, specifically, and constructively the REC conveys its decisions and feedback in the decision letter. This includes explaining the rationale for each concern, formulating firm and actionable recommendations, and directing applicants to relevant local, national, or international guidelines and documents that can help them appropriately revise their submission (Clapp et al., 2017; Sidaway et al., 2023). A clear communication style improves stakeholder engagement, sets realistic expectations, preserves resources, and helps reduce turnaround times for both RECs and investigators.

Kyrgyzstan, like many of its regional post-Soviet, post-communist counterparts, faces significant structural and systemic challenges. These include limited financial and human resources, ageng health infrastructure, and fragmented governance, factors that collectively pose barriers not only to public health preparedness, but also to the integrity and responsiveness of ethical review systems (Kudaibergenova et al., 2023; Semenova et al., 2024). By examining the institutional capacity of RECs in this context, we seek to contribute to a better understanding of the operational realities faced by ethical oversight bodies in LMICs, and to inform future efforts aimed at strengthening their role in public health research governance.

## Methods

A retrospective, single-center analysis was conducted on REC decision letters related to study protocols submitted for ethical review between January 2018 and December 2022.

### Selection of REC

Our analysis focused on the REC affiliated with the Institutional Editorial Office of the scientific and practical journal *Health Care of Kyrgyzstan*, under the Ministry of Health of the Kyrgyz Republic. The REC was created in 2003 at the Scientific and Production Association "Preventive Medicine" of the Ministry of Health. In 2023, the REC was transferred to the Institutional Editorial Office. The REC was selected for its status as the longest standing REC in the country, with over 20 years of continuous operation and a specific focus on public health research. It also processes the highest volume of public health protocol submissions nationally and is registered with the U.S. Office for Human Research Protections (OHRP; IORG#0008909).

### Standard Operating Procedure of the REC

In Kyrgyzstan, the standard operating procedures (SOPs) of RECs are relatively uniform across institutions. This is because in Kyrgyzstan, the REC SOPs are contextualized via the Eurasian Economic Union (EAEU) framework. For the study period 2018–2022, the applicable EAEU framework was the Eurasian Economic Commission (EEC) Council Decision No. 79 (dated 3rd November 2016), which describes the Rules of Good Clinical Practice for the EAEU member countries. This framework, in its annex, provides a supranational baseline that contributes to the relative degree of SOP standardization among RECs within the member states (Eurasian Economic Commission, 2016).

The REC operational process begins with a preliminary review by the REC Secretariat to verify the completeness and accuracy of the submitted documentation. If all required forms and details are in order, the protocol is formally logged in the REC registry under “Received/Submitted Protocols”. At this stage, the date of submission, protocol title, and the applicants’ name are recorded, and a unique REC serial number is assigned. Following this, the documents are forwarded to the REC Chair, who determines the suitability for review.

If deemed suitable, the Chair assigns one or more qualified REC member(s) as primary reviewer(s). The Secretariat sends the application to the designated primary reviewer(s), who conducts an initial assessment of the protocol. During the REC meeting, the primary reviewer(s) presents a summary of the protocol, either orally or in writing, along with their evaluation (Box 2). The Chair then facilitates a discussion of the full submission package, which typically includes the study protocol, investigators’ brochure, informed consent form, investigator and site qualifications, and any advertising materials.

Box 2. Evaluation form provided to the primary REC reviewer(s) for assessing the application.

When conducting the review, attention should be paid to the following criteria^a^:

 □ Minimize the risk to study participants
 □ Risk to participants must be reasonable compared to the expected benefit
 □ Selection of participants should be uniform and unbiased
 □ Informed consent must be clear, understandable, and properly completed
 □ Study plan should provide adequate conditions for monitoring the collected data for the safety of participants
 □ There should be adequate conditions for the protection of privacy, maintaining confidentiality where necessary
 □ Appropriate measures are in place to protect vulnerable participants
 □ If necessary, recording of comments
 □ Signature of the assessor and date

^a^The completed evaluation form is an official confidential record of the decision made by the REC.

Recommendations for changes to the protocol, associated forms and/or advertising materials are recorded in the meeting minutes and sent to the study applicant. Post-discussion the chair announces a vote with four possible outcomes:

- *Approved with no further recommendations*. The study documents are approved as such without need for modifications.
- *Provisionally approved with recommendations*. The study documents are approved in principle and does not require another full round of review, provided that the concerns raised are adequately addressed by the study team. The revised documents, whether amended or supplemented, must be submitted to the REC for a final approval by the Chair.
- *Require re-consideration*. The study may be granted approval following another round of full review by the REC members. The study team is required to provide detailed clarifications, modifications, or rebuttals to the queries raised by the REC.
- *Reject*. The study protocol has been deemed unsuitable for consideration and implementation due to significant concerns. The REC provides a detailed rationale for its decision to reject the proposal.

The decision is recorded in the REC logbook along with the date of decision. If the REC approves or provisionally approves the protocol, the Secretariat sends the applicant a decision letter along with the requested changes (if) and required checkpoints for verification of the investigators’ fulfillment of ethical and moral obligations throughout the study. On the other hand, if the REC votes to reject the application, the Secretariat informs the applicant in writing of the decision taken and the reasons for rejecting the application. The study team has the opportunity to appeal by contacting the Secretariat.

### Data Collection and Analysis

We obtained copies of REC decision letters and corresponding study protocols from the REC's official archive. Access to the sensitive REC documents and records was granted after formal approval from the REC chairperson and leadership. The documents were never removed from the archive room, with the approval granting access solely for the purpose of this study. All local and institutional regulations were complied with, including necessary ethical oversight. Confidentiality of the documents and its contents was always maintained, and no identifiable information was disclosed. Importantly, the REC chairperson and leadership were not involved or consulted in the study design, methodology, data collection and analyses, manuscript preparation, and the decision to publish the results.

All documents were translated to English by the study authors and assigned a random serial number. From the decision letters, we manually extracted key data, including the date of submission, type of processing requested (expedited or regular), number of REC members involved, their gender, educational background, and institutional affiliation. We also recorded the final decision, the date of decision, and any recommendations provided by the REC. For the documentary review, all REC decision letters were text mined to assess the nature and scope of REC observations, as well as the perceived relevance and clarity of the recommendations from the perspective of the study authors. Quantitative data were organized and stored using Microsoft Excel 365 (Windows), and descriptive analyses were conducted using R version 4.4.0.

## Results

A total of 138 applications were submitted to the REC for review and approval. Among these, 49 (36%) were submitted during the pre-pandemic period, and 89 (64%) during the pandemic period. No submitted application was excluded from our analysis. Except for three applications reviewed via online meetings, all decisions were made during in-person sessions. Expedited processing was requested for 34 applications (25%), including 14 from the pre-pandemic period and 20 during the pandemic period.

### REC Composition

The number of REC members involved in reviewing and deciding on each application ranged from five to nine, with most applications reviewed by panels of seven members (Table 1). However, data on the identity of 51% (*n* = 506/991) of REC members were not available in the records. Importantly, each REC panel comprised of one chairperson and one secretary. In most cases, the final approval letters mentioned the number of panel members but did not provide further details. Among the members for whom identifiers were available, 46% were female and 3% male. Most of the identifiable members had a background in public health, with 17% working as epidemiologists and 6% as hygienists. Legal professionals represented the second most common background, accounting for 14% of members. In terms of institutional affiliation, 40% of REC members worked at local academic institutions, 6% were affiliated with non-governmental organizations, and 3% were self-employed.

**Table 1.** Overview of the Research Ethics Committee (REC) Composition.

REC Composition^a^	Pre-COVID Pandemic (Years 2018–2019)	During COVID Pandemic (Years 2020–2022)	Total
*Number of members per application (n = 138 applications)*			
5	0 (0%)	23 (16%)	23 (16%)
6	11 (8%)	4 (3%)	15 (11%)
7	30 (22%)	14 (10%)	44 (32%)
8	0 (0%)	26 (19%)	26 (19%)
9	8 (6%)	22 (16%)	30 (22%)
*Sex (n = 991 REC members)*			
Male	8 (1%)	21 (2%)	29 (3%)
Female	152 (15%)	304 (31%)	456 (46%)
No Information	188 (19%)	318 (32%)	506 (51%)
*Professional background (n = 991 REC members)*			
Biomedical	8 (1%)	21 (2%)	29 (3%)
Legal and Paralegal	49 (5%)	89 (9%)	138 (14%)
Public Health (Epidemiologist)	59 (6%)	110 (11%)	169 (17%)
Public Health (Hygienist)	19 (2%)	42 (4%)	61 (6%)
Sociologist, Ethicist, Philosopher	8 (1%)	21 (2%)	29 (3%)
Accountant	17 (2%)	42 (4%)	59 (6%)
No Information	188 (19%)	318 (32%)	506 (51%)
*Institutional affiliation (n = 991 REC members)*			
Kyrgyz academic institution	134 (14%)	260 (26%)	394 (40%)
Kyrgyz non-governmental	19 (2%)	41 (4%)	60 (6%)
Self-employed	8 (1%)	22 (2%)	30 (3%)
No Information	187 (19%)	320 (32%)	507 (51%)

a Note that the same reviewer (committee participant) may have participated in evaluation of multiple different REC applications. The aggregated metrics provided here do not account for such instances since individual information was not ascertainable in all cases.

### REC Review Outcomes

In the initial review, REC members approved 76 applications, resulting in an approval rate of 55.1% (Table 2). Three applications required reconsideration, with the REC requesting improvements to the study design in all cases. Among these, one application was resubmitted and subsequently approved; another received provisional approval in the second round; and the third was never resubmitted. The remaining 59 applications (42.8%) received provisional approval in the first round, with recommendations to strengthen the study protocol. Among the recommendations provided for approved applications, the most frequent concerned data collection, protection, and storage (46%), followed by participant protection and rights (27%), and study design (27%; Table 3). Importantly, no application was rendered a reject decision across the study period.

**Table 2.** Research Ethics Committee (REC) Decision Based on the Type of Application (*n* = 138).

REC Decision	Pre-COVID Pandemic (Years 2018–2019)	During COVID Pandemic (Years 2020–2022)	Total
*Approved at first submission with no further recommendations (n = 76)*			
Expedited applications	6	15	21
Regular applications	18	37	55
*Provisionally approved with recommendations (n = 59)*			
Expedited applications	8	4	12
Regular applications	16	31	47
*Require re-consideration (n = 3)*			
Expedited applications	0	1	1
Regular applications	1	1	2

**Table 3.** Domain of Improvement for Provisionally Approved Applications at First Submission with Recommendations (*n* = 59).

Domain of Improvement	Pre-COVID Pandemic (Years 2018–2019)		During COVID Pandemic (Years 2020–2022)		Total
	Expedited	Regular	Expedited	Regular	
Study design	1	3	1	11	16 (27%)
Participant protection and their rights	2	3	0	11	16 (27%)
Data collection, analysis, and storage	5	10	3	9	27 (46%)
Total	8 (14%)	16 (27%)	4 (6%)	31 (53%)	59 (100%)

### REC Review Time

**Figure 1.**
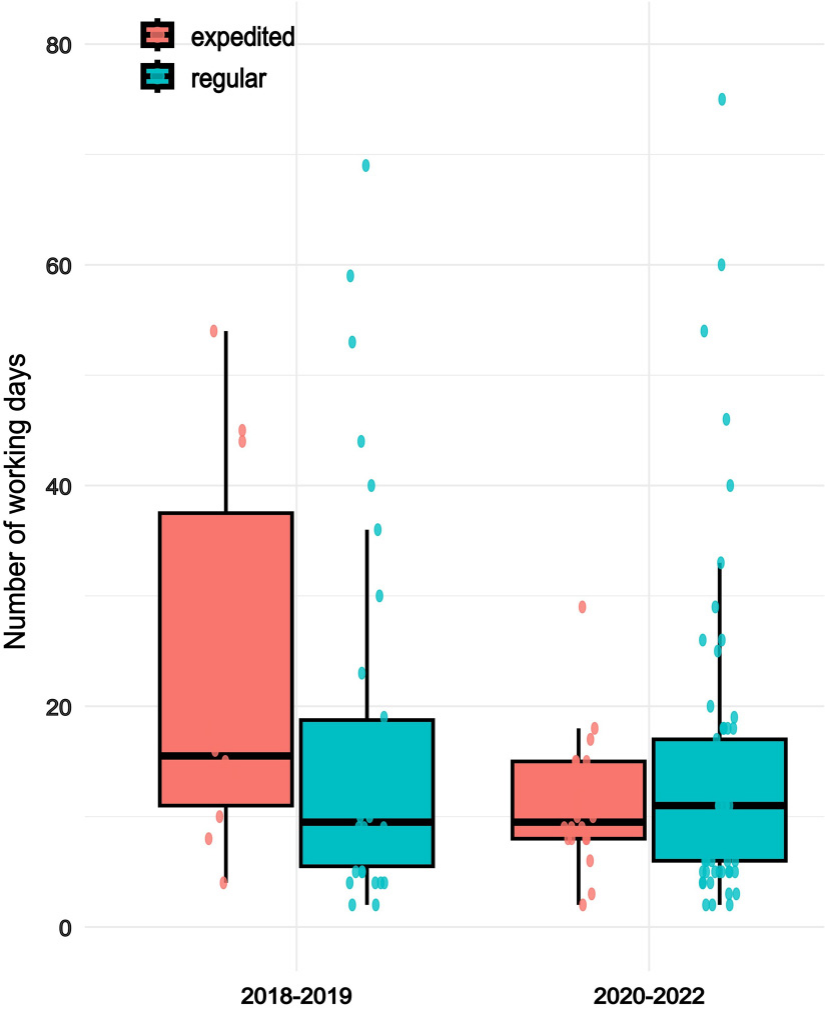
Distribution of time (in number of working days, excluding weekends) from the date of REC protocol submission to the date of final decision. Date of final decision does not represent the date when the decision was handed to the research teams. Red represents expedited protocols, and blue represents protocols processed through the regular review pathway. Data was missing for five protocols from the 2018–2019 period–four expedited protocols and one regular processing protocol.

The median review time, defined as the duration from date of submission to date of REC decision (excluding weekends), for expedited protocols was 15.5 days (Q1 to Q3; 11 to 37.5) in the pre-pandemic period which reduced to a median of 9.5 days (Q1 to Q3; 8 to 15) during the COVID-19 pandemic. Conversely, for regular protocols, the median time increased from 9.5 days (Q1 to Q3; 5.5 to 18.8) in the pre-pandemic period to 11 days (Q1 to Q3; 6 to 17) during the pandemic (Figure 1).

### Documentary Review

To complement our quantitative findings, we conducted a text-mining analysis of the REC applications and decision letters to generate a documentary review. This exercise provided greater insight into the nature of the deficiencies identified by the REC and the extent of the revisions requested from the research teams (Table 4). It allowed us to identify critical gaps and ambiguities in REC guidance and recommendations that could be addressed in future decision letters for enhanced clarity for the applicants.

**Table 4.** Remarks from Select REC Decision Letters That Were Mined for Documentary Analysis Including Observations and Recommendations Made by the REC.

Letter Serial	Domain of Improvement^a^	Observations^b^	Recommendations^b^
#K-RP09-1 (initial submission)	Study design	The research protocol does not comply with good international practices and does not contain a detailed description of the procedures for observing ethics in social research. The assessment of compliance with ethical issues had to be assessed by indirect methods. One of the appendices does not correspond to the study.	Recommend that the research protocol be drawn up more efficiently and in line with good practices. The protocol should indicate the name of the study, describe procedures for maintaining confidentiality and obtaining informed consent and other issues of compliance with ethical standards, rules for storing and publishing data.
#K-RP07-2 (revised submission)	Participants and their rights	The study protocol submitted for re-examination contains corrections. In the submitted version, the title of the protocol and the purpose of the study correspond to each other. The numbering of the sections of the protocol has been corrected. An explanation has been added to the protocol that healthcare workers will be interviewed. Contradictions in ethics between the content of the study and the description of the procedures have been addressed. The researchers have attached the previously missing informed consent forms and data confidentiality forms. But the question arises as to how healthcare workers will be interviewed since there are no tools specified for interview process. There is no description of compliance with ethical issues for healthcare organizations participating in this study, which creates certain ethical risks and conflicts of interest, since the research team has employees of the respective healthcare organization.	The research team is asked to explain what questionnaire or interview tools will be used for healthcare workers. To avoid ethical risks and conflicts of interest, the REC recommends that the following be included in section “*Potential risks*” - all information will be analysed and included in reports only in a generalized form. Responses in the final report will not be linked to names or affiliations with individual healthcare organizations. Furthermore, authors must acknowledge in protocol that workplace associated gaps identified because of their study cannot be used for punishment or disciplinary or administrative measures. The research team guarantees to use these results in aiding healthcare organizations to develop an action plan to eliminate the identified gaps and improve the healthcare delivery system in the healthcare organizations that participated in the study. Finally, authors must ensure that the results of the assessment in individual healthcare organizations cannot be transferred to supervisory and/or control organizations to avoid ethical risks.
#K-RP05-1 (initial submission)	Data collection, analysis, and storage	The protocol does not describe procedures for maintaining confidentiality and anonymity of personalized data storage. Safe conditions for data storage and analysis, as well as procedures for dissemination and use of results are not specified. Persons with access to the data are not defined.	Participants’ data privacy must be protected throughout the survey and data analysis period.

a Only primary domain of improvement has been considered in our study. We acknowledge that a study could have been deficient under multiple domains and REC could have requested clarifications and modifications under multiple domains.

b Observations and recommendations have been translated from Russian to English by the study authors who are proficient in both languages. Mild grammatical and syntactical changes have been incorporated to enhance interpretation for English readers without changing the intended meaning. Consequently, backward translation was also performed using Google Translate to ensure accuracy in translation. Study authors were not part of the REC commissions or deliberations and hence are not responsible for the content of the REC decision letters and as such cannot be held liable in this regard.

We observed that the REC decision letters contained deficiencies that could be grouped into three broader domains. For instance, the REC often failed to explicitly identify or request the correction of common shortcomings in submitted protocols. Protocols lacked sufficient detail regarding the number of research team members, their professional backgrounds, and their specific roles within the study. While sample size calculations were included in most protocols, it was unclear whether these had been developed in consultation with a statistician or an individual with relevant statistical expertise. Information on funding sources or financial support was also frequently omitted. Similar gaps were observed in data management planning. Many protocols did not adequately address key elements such as anonymization procedures, access controls, or data storage protocols. Furthermore, the REC did not routinely request clarification on the intended end use of the study findings i.e., whether for publication, internal evaluation, educational purposes, or other uses.

As noted, the names and educational background for most of the REC panel members were missing. We also believe that the recommendations provided by the REC were vague and lacked actionable specificity. For example, the REC frequently requested the study teams to comply with good international ethical practices but failed to cite any national or international guidelines (such as the CIOMS 2016 Guidelines (Council for International Organizations of Medical Sciences, 2016) or the relevant versions of the Declaration of Helsinki (World Medical Association, 2025)) that the investigators could reference. No practical tools, checklists, or templates were developed or recommended by the REC to assist the investigators in preparing an ethics and data monitoring plan, a task that could be particularly challenging in studies involving vulnerable patient populations or first-time principal investigators.

## Discussion

To our knowledge, the present study reports the first assessment of REC activities in Kyrgyzstan, highlighting both strengths and critical gaps in the ethical review of public health research. While previous studies in post-communist countries have documented challenges related to organization, policy, training, and resource allocation (Aniyozova & Strosberg, 2024; Famenka, 2011, 2016; Sarvmsakova, 2009; Strosberg et al., 2013), our approach goes further by exploring the underlying causes of these issues. We identify transparency, quality, and communication style as key factors potentially affecting the understanding and relationship between RECs and researchers submitting research protocols. Our study also demonstrates the utility of REC decision letters as a valuable and under-utilized data source for evaluating these dimensions, especially in LMIC contexts.

One of the first deficiencies we identified was the absence of identifying details for 51% of REC members who participated in reviews. This omission likely stems from the lack of uniform standards for drafting REC letters, resulting in considerable variability in both structure and content. Across many CIS countries, REC communications are issued as extracts from meeting minutes and prepared according to general administrative guidelines, since no specific legislative requirements exist that standardizes the format. Under these circumstances, each institution adopts its own internal practices, guided by broad principles for official records. Typically, meeting minutes list all attendees or, when participant numbers are large, attach a separate appendix, noting only the total number of participants in the main text. These minutes and the appendix are then signed by the chair and sealed by the institution stamp.

In the REC we reviewed, the absence of attendee names in its extracts appears to reflect a local practice, *potentially out of convenience*, that directly contradicts its own SOPs, which mandates an appendix of members present and invited. This practice also violates multiple international guidelines (Table 5) which clearly mandate the documentation of REC panel composition as a core element of its validity, legitimacy, and accountability (African Bioethics Consortium, 2017; Council for International Organizations of Medical Sciences, 2016; World Health Organization, 2000). A previous study from India, analysing REC letters from 2006 to 2009, found that 20% of the letters did not include the names of panel members, with this figure rising to 45% when considering the absence of their designations (Taur et al., 2011). The omission of such information in REC documentation impairs ones’ ability to fully reconstruct the decision-making process and limits the possibility of independent verification or reproducibility. This, in turn, undermines confidence in and accountability of the ethical review process, particularly when decisions involve vulnerable populations or socially sensitive issues.

**Table 5.** Mandatory Requirement of Documenting Identifiers for REC Panel Members Involved in the Review Process According to International Guidelines.

Document	Document Abstract with Section Number
World Health Organization (WHO) Operational Guidelines for Ethics Committees that Review Biomedical Research, 2000	**Section 4.7. Record keeping** – The minutes of the meeting should clearly record the members present, the quorum, decisions taken, the rationale for decisions, and any dissenting opinions.
	**Section 4.5. Quorum requirements** – indicate the minimum number of people present for a quorum; record the professional profile of the members (medical, legal, representative of the public).
Council for International Organizations of Medical Sciences (CIOMS) Guidelines, 2016	**Guideline 23** – The composition and functioning of ethics committees should be transparent, with written procedures and documentation of deliberations.
Research Ethics Committee Assessment Toolkit (RECAT), 2017	**Section 3.2. Committee Functioning** – Documentation of committee membership and attendance at each meeting is essential to demonstrate quorum and ensure that decisions are made by appropriately constituted bodies.

Nonetheless, among the participants with identifiable information, we observed a strong predominance of female colleagues. This can be attributed to the country's demographic and socio-economic context. Women make up over 50% of the population and have traditionally been employed in sectors such as education, health care, and social services, fields that frequently intersect with ethics review processes. According to the 2023 National Statistical Committee of the Kyrgyz Republic, women constituted 77.4% of the workforce in education and 78.1% in health care and social services (National Statistical Center of the Kyrgyz Republic, 2024). Such patterns are also likely shaped by the Soviet legacy and persistent gender inequality in economic parity. These sectors, although essential, are also among the lowest paid in the post-Soviet region and have historically been dominated by women. A similar trend has been reported in neighboring Kazakhstan, where it was found that the RECs consisted of 111 members, with 69% (77) of the members being women and remaining 31% (34) being men (Fakhradiyev et al., 2024).

Next significant observation was the exclusion of lay persons in REC panel compositions. According to WHO's Standards and Operational Guidance for Ethics Review of Health-Related Research, a lay person is defined as someone without professional training in biomedicine, clinical research, or related disciplines, and who is not affiliated with a research institution (World Health Organization, 2011). Lay members are expected to represent the interests of potential research participants and contribute to discussions on ethical procedures, the clarity of presented information, voluntariness of consent, and protection of rights. Their absence in a REC weakens its ability to effectively account for public interests, resist institutional bias, and protect vulnerable groups, especially in public health research which often involves social risks (Famenka, 2011). Such instances also raise concerns about transparency, as professional homogeneity can limit critical scrutiny of research protocols.

More recent WHO guidance, particularly the Tool for Benchmarking Ethics Oversight, stresses that participation of lay members should not be merely symbolic. There should be enough lay members to meaningfully influence discussions and decisions (World Health Organization, 2023). These principles are also described in the CIOMS guidelines and the International Council for Harmonisation–Good Clinical Practice (ICH-GCP) standards, which emphasize the value of including members with non-medical or non-academic backgrounds to broaden perspectives and better reflect public concerns (Council for International Organizations of Medical Sciences, 2016; International Council for Harmonisation of Technical Requirements for Pharmaceuticals for Human Use, 2025). Empirical studies too support these recommendations, showing that lay members improve discussion quality, increase sensitivity to cultural and social contexts, and build participant trust (Fakhradiyev et al., 2024; Ganesh & Kalikar, 2025; Nicholls et al., 2023; Solomon, 2016). A review of our REC's SOPs showed that they do mandate the inclusion of at least one lay person–described as “*… at least one specialist whose main area of activity is not the field of medical science…*”.

Although, the current absence of lay members raises concerns about adherence to the established SOPs, it may reflect a broader regional pattern. In Kazakhstan, for example, surveys have revealed that while lay members are included (Fakhradiyev et al., 2024), their involvement is often inconsistent and informal. The literature identifies several reasons for such behavior. Researchers have highlighted that the concept of a “lay member” is often poorly defined, leading to ambiguity and tension within RECs (Emmerich, 2009). The involvement of non-experts may be perceived as a challenge to professional autonomy or as a destabilizing influence, particularly when their input questions the scientific validity of research protocols (Humphreys et al., 2014). Such issues are compounded in low-resource settings where RECs do not have experience of effectively integrating lay members and do not offer adequate support mechanisms for their training and inclusion (Green, 2007).

Furthermore, studies have shown that lay members often lack clarity about their role, while other committee members may not be fully cognizant of the value they bring (Legood, 2005). As a result, their contributions are frequently overlooked or limited to commenting on the language of information sheets. This devalues their contributions and often reduces adherence to guidelines into a mere formality, or worst leads to their exclusion altogether (Solomon, 2016). In RECs, that tend to operate within closed expert circles, external voices are perceived not as assets but as irritants or even risks. Such views may be rooted in local regulations or shaped by historical institutional practices common in post-communist countries.

Our reviewed REC had a higher-than-expected rate of approvals at first review, which was largely driven by a notable increase in first-review approvals for expedited protocols during the pandemic. These findings align with reports from a Spanish REC, which also documented a doubling in the proportion of favorable outcomes–approval with no or minor recommendations–granted at first review during the pandemic (Mirpuri et al., 2022). When comparing approval rates by type of protocol processing, a study from South Africa reported that 37% of expedited protocols and 18% of regular protocols were approved at first review without further recommendations (Cleaton-Jones & Grossman, 2015). In contrast, during the pre-pandemic period, our reviewed REC approved 43% (6/14) of expedited protocols and 51% (18/35) of regular protocols at first review without additional recommendations. These numbers rose to 75% (15/20) for expedited protocols and 54% (37/69) for regular protocols during the pandemic, indicating a comparatively higher rate of initial approvals across both categories.

Interestingly though, the overall first-review approval rate at our REC, regardless of the time period or type of processing, was substantially higher at 55.1%, compared to RECs from other countries including 4% in Finland (Happo et al., 2016), 24% in South Africa (Cleaton-Jones & Grossman, 2015), 28% in the United Kingdom (Dixon-Woods et al., 2016), 31% in France (Decullier et al., 2005), and 38% in Spain (Mirpuri et al., 2022). Several factors may contribute to this higher rate. Notably, all submissions are thoroughly screened by the REC Secretary prior to being logged in the database. Applications are only accepted once all required documents are complete, properly annexed, signed, and accounted for. This strict pre-review screening process likely reduces the number of rejections or requests for additional documentation after formal REC review. Supporting this, among the 138 applications reviewed during the study period, only one application received a REC notification due to a missing patient consent form.

Second, the reviewed REC may face unique challenges regarding the thoroughness of its analysis of the submitted research protocols. This concern is supported by our documentary analysis, which identified several deficiencies that were not flagged during the REC's review process. Regional studies have also reported similar significant gaps in the depth and rigor of ethical review conducted by RECs (Fakhradiyev et al., 2024). We believe this issue may stem from a lack of necessary expertise among panel members, compounded by the absence of robust national programs for the protection of human research participants. As a result, critical ethical aspects may have been overlooked, leading to the approval of applications that warrant further scrutiny. Finally, another contributing factor may be the nature and complexity of the protocols submitted for review. Among the 138 applications analysed, approximately 70% were observational non-interventional studies, while only four were clinical trial protocols. The predominance of low risk, less complex studies may have contributed to limited experience in handling more complex protocols, potential leniency in the review process, and consequently, higher first-review approval rates and faster turnaround times.

The median review times for both regular and expedited protocols at the studied REC were notably shorter than those reported in many other settings. For example, Tanzania reported a median turnaround time of 32 days (Mrisho & Essack, 2021) the United States 286 days (Greene & Geiger, 2006), and a South African university REC reported an average of 105 days (Clarke, 2014). However, a study from a REC in Saudi Arabia, with comparable workload, reported a median turnaround time of 7 days for expedited protocols and 20.5 days for regular protocols (AlFattani et al., 2023), closely aligning with the review times observed at our REC. As seen globally, the COVID-19 pandemic led to a sharp increase in protocol submissions, many requiring expedited review. A similar surge was observed at the studied REC. In response, the committee managed to reduce the median turnaround time for expedited reviews from approximately 15.5 days to 9.5 days (excluding weekends), demonstrating an ability to adapt under pressure. Comparable trends were reported by authors from Kenya, where the median review time for expedited protocols dropped from 93 days before the pandemic to 59 days during the pandemic. A slight improvement in median processing time for regular protocols was also reported by the study authors, from 89.5 days pre-pandemic to 83.5 days during the pandemic (Kebenei et al., 2024).

Nonetheless, two notable findings emerged from our REC data. First, the median review time for expedited protocols before the pandemic (15.5 days) was unexpectedly longer than that for regular protocols (9.5 days), contrary to typical expectations. This may be a statistical artifact, as the REC publishes its quarterly meeting schedule in advance, allowing researchers to time submissions accordingly and potentially reducing the apparent turnaround time for regular protocols. In contrast, expedited reviews requires assembling a specific panel, including a research fellow, a non-research fellow, a subject-matter expert, a physician, and an external reviewer. Coordinating the availability of all members likely introduced delays, contributing to the paradoxically longer processing time for expedited protocols.

At the same time, we cannot entirely dismiss the possibility of inconsistencies in internal prioritization or workflow processes of the REC, particularly in the classification and handling of expedited versus regular reviews. Applications were deemed eligible for expedited review by the REC simply because their submission coincided with the REC's resumption of activity after an extended summer recess, or when the study team reported time constraints linked to funding deadlines. The second standout observation was that unlike the global trend of reduced review times during the pandemic, our REC experienced a slight increase in the median review time for regular protocols, from 9.5 days pre-pandemic to 11 days during the pandemic. This could be due to shifting prioritization and resource allocation towards expedited protocols during the pandemic (Kornioti et al., 2024). Furthermore, unfamiliarity with remote online working and resulting administrative disruptions could have also led to the observed delays (Kadam et al., 2022).

We also identified three interconnected and interdependent issues in REC's decision letters including insufficient quality of review, lack of transparency in REC documentation, and inadequate communication of reliable information by REC. These issues may reflect broader systemic challenges common in ex-Soviet countries, where national research ethics systems often remain underdeveloped. Apart from the factors discussed above, issues such as inconsistent standards of ethical review across different types of biomedical research, limited independence, and varying levels of institutional capacity to ensure the protection of human research participants, precipitate the situation.

A study from Kazakhstan found that only 25% of RECs had a dedicated annual budget and administrative staff, resulting in a score of just 56.2% in the “Resources” dimension. The “Review of Individual Protocol Items” dimension was rated even lower, at only 33.7%, highlighting significant concerns about the depth and quality of ethical review (Fakhradiyev et al., 2024). Similar trends were reported in a multinational survey of 64 RECs across three LMICs–Egypt, India, and South Africa. The survey found that only a third of RECs had an annual budget, 28% had a quality improvement program, 40% required mandatory ethics training for REC members, and 47% conducted continuing education activities (Silverman et al., 2015). These findings underscore persistent gaps in infrastructure, capacity-building, and quality assurance across RECs in LMIC settings.

At the same time, it is important to enhance the knowledge and competencies of REC members–not only in ethical review but also in scientific evaluation. Studies have suggested that REC members often struggle with reviewing and evaluating the alignment of study design with available literature, research objectives, and statistical methods, especially as research designs become increasingly complex (Ainembabazi et al., 2021; Hunt et al., 2016). This gap in scientific scrutiny may compromise the reliability of the findings, potentially exposing participants to avoidable risks and undermining the integrity of studies. Addressing this issue requires the development of more robust mechanisms for the recruitment, selection, and continuous training of REC members.

In the reviewed REC, training of panel members in ethical review is clearly defined as a REC responsibility. As an internal division of the Institutional Editorial Office, the REC operates under the institutional charter and is governed by the Code of the Kyrgyz Republic No. 23, “*Labor Code of the Kyrgyz Republic*” (dated January 23, 2025) (Supreme Council of the Kyrgyz Republic, 2025). According to its SOPs, the REC is required to implement partial rotation of its members after a three-year term and is encouraged to maintain continuity by establishing a dedicated training institute for REC personnel. Additionally, eligibility criteria for REC membership include a documented “*… readiness to follow the rules of Good Clinical Practice (GCP) …*”, emphasizing adherence to internationally recognized ethical standards.

Furthermore, during the COVID-19 pandemic, the REC had actively participated in the “International Postgraduate Training Program in Bioethics Research–Central Asia Network,” supported by the Fogarty International Center and the National Institutes of Health (NIH). As part of this initiative, several REC members completed onsite or online training between 2021 and 2024 at Harvard Medical School and Boston Children's Hospital. In addition, the REC organized several international conferences in Kyrgyzstan in 2023, in collaboration with both institutions, further contributing to capacity-building and regional knowledge exchange in research ethics. Since the trainings started in 2021, future research on REC operations, including in-depth comparison of decision letters, could provide valuable insights into the programs’ impact by assessing how acquired knowledge and training translates into practice.

Clearly, there is a need to develop and implement uniform standards for the design of REC documentation, including mandatory elements in protocol extracts. This is especially important for increasing trust in RECs, given the growing role of bioethics and public oversight in clinico-social research within the field of public health in LMICs, including Kyrgyzstan. Accordingly, we propose the following recommendations:

- Develop a clear definition of a lay member, including their roles, responsibilities, and procedures for recruitment and appointment. In addition, legal provisions at the national level should mandate the inclusion of lay members in REC deliberations.
- Take measures to enhance the thoroughness of protocol review by REC members. Committees should adopt standardized checklists and review forms to ensure systematic evaluation of all critical aspects of research protocols. It is essential that these tools are tailored to different study designs and include clear criteria for identifying protocols eligible for expedited review.
- Design and implement mandatory training programs to build both scientific and ethical competencies among REC members. Training should include topics such as risk assessment, research methodology, basics of statistical analysis, and ethical considerations specific to vulnerable populations.
- Strengthen regulatory oversight through the establishment of a national accreditation system for RECs, overseen by a central authority or national ethics commission. This body should harmonize SOPs, conduct regular audits, issue guidance, and maintain an official registry of all ethics committees.
- Increase resources allocated for the effective functioning of RECs. Governmental and institutional support is essential to ensure adequate funding, administrative capacity, and infrastructure. Dedicated budgets would allow RECs to hire qualified staff and provide ongoing professional development.
- Promote international collaboration to support knowledge exchange and capacity-building. Partnerships with international organizations and experienced RECs from other countries can facilitate the sharing of best practices and innovative approaches to ethical review.
- Implement continuous quality improvement measures through regular self-assessment using standardized tools to identify gaps and monitor progress over time.

For implementation of these recommendations, we suggest a multi-level approach. The recommendations should be discussed at all levels of policy development, from the Ministry of Health to national regulatory bodies responsible for public health research oversight, to the RECs themselves. Such an approach will ensure consistency, avoid excessive burden and responsibility on a specific institution, accommodate diverse viewpoints within different levels of the system, and allow for institutional flexibility.

Finally, we would like to contextualize our findings in the background of some methodological limitations of our study. This was a single center analysis and future research is needed across other RECs in the country and the wider CIS region. Also, we did not collect qualitative empirical data from REC members, applicants, or other stakeholders (via structured interviews or surveys). Consequently, our interpretations of transparency, review quality, and communication style are inferred from REC decision letters alone which may not fully capture the intentions, constraints, or informal practices that may shape REC decision-making in routine practice. Lastly, we defined transparency, review quality, and communication style subjectively based on the information that is expected to be specified on the REC decision letters based on its template documents. These constructs have not been validated externally, which may limit comparability with other studies and introduce some subjectivity into our assessments.

Future work in this area should undertake comparative studies of RECs functioning across institutions and study domains to identify best practices and variabilities in SOPs implementation. We also recommend assessment of ethical review in non-clinical public health research, addressing the documented asymmetry where clinical trials are regulated but community-based or observational studies are under-regulated (Kudaibergenova et al., 2023). Once national-level interventions are developed and implemented, we recommend a re-assessment of their impact on REC performance while also seeking stakeholder viewpoints for refining the system further. Finally, stakeholder perspectives and their familiarity with the international best practices needs assessment, including researchers and community representatives, to improve transparency, communication, and overall ethical oversight.

In conclusion, while the RECs in Kyrgyzstan demonstrate some capacity to conduct ethical review during public health emergencies, there are identifiable gaps in transparency, review quality, and communication practices. Consequently, there is an urgent need to develop recommendations for establishing a national programme for ethical regulation of public health research. Such measures will help foster a culture of continuous improvement and responsiveness to evolving ethical standards. By implementing the recommended actions, RECs can enhance their effectiveness and align with global efforts to uphold high ethical standards in research. Strengthening RECs will not only protect the rights and welfare of research participants but also improve the credibility and overall quality of research conducted within the country.

## Best Practices

Standardized decision letters with plain-language templates and checklists should be developed and adopted that explicitly require citation to national and international guidance. RECs should train members to provide feedback that is specific, actionable, and proportionate, clearly distinguishing mandatory stipulations from recommendations. Regulatory oversight committees should be established and should routinely monitor and share core quality metrics including turnaround time, first-pass approval rates, and recurrent deficiencies, to guide improvement. Finally, RECs should work towards strengthening capacity with the inclusion of lay/public members and members with non-biomedical perspectives.

## Research Agenda

REC decision letters serve as the primary interface between RECs and study investigators. The clarity, content, and style of communication in decision letters shape participant protections, investigator burden, and trust in ethical review process. Yet evidence from LMICs and post-Soviet settings regarding communication depth in REC decision letters remains scarce. We addressed this gap by analysing all decision letters (*n* = 138) from a public-health REC in Kyrgyzstan, from 2018 to 2022, with an aim to evaluate transparency, review quality, and communication, including guideline citation, turnaround time, and first-pass approvals, to inform practical and actionable insights and recommendations.

## Educational Implications

Our study shows that the RECs in Kyrgyzstan, and possibly in other similar settings, may not be adequately prepared to provide ethical oversight for public health research, especially during emergencies. We identified key shortcomings in areas of transparency, quality of expertise, and communication practices. There is a pressing need for a comprehensive system of ethical standards, serving as a foundational stone for developing a national oversight program. Central to this program must be the principle of community engagement in the decision-making process, in alignment with local needs and contexts. Such a framework would also ensure the effective protection of research participants at both the individual and population levels.

## References

[bibr1-15562646261428333] African Bioethics Consortium. (2017). Research Ethics Committee Assessment Toolkit (RECAT). Version 1.0. Johns Hopkins University, Baltimore Maryland USA. [online]. Retrieved August 7, 2025, from https://bioethics.jhu.edu/wp-content/uploads/2018/12/RECAT-Toolkit.pdf

[bibr2-15562646261428333] Ainembabazi P. Castelnuovo B. Okoboi S. Arinaitwe W. J. Parkes-Ratanshi R. Byakika-Kibwika P. (2021, September 26). A situation analysis of competences of research ethics committee members regarding review of research protocols with complex and emerging study designs in Uganda. BMC Medical Ethics, 22(1), 132. 10.1186/s12910-021-00692-6 34565355 PMC8474911

[bibr3-15562646261428333] AlFattani A. AlBedah N. AlShahrani A. Alkawi A. AlMeharish A. Altwaijri Y. Omar A. AlKawi M. Z. Khogeer A. (2023, July 10). Institutional review boards in Saudi Arabia: The first survey-based report on their functions and operations. BMC Medical Ethics, 24(1), 50. 10.1186/s12910-023-00928-7 37430255 PMC10334562

[bibr4-15562646261428333] Aniyozova D. Strosberg M. A. (2024, April 17). Ethics review of biomedical research in Uzbekistan: Policy and program gaps. Asian Bioethics Review, 16(4), 653–661. 10.1007/s41649-023-00273-2 39398450 PMC11464834

[bibr5-15562646261428333] Burgess T. Rennie S. Moodley K. (2023, February 15). Key ethical issues encountered during COVID-19 research: A thematic analysis of perspectives from South African research ethics committees. BMC Medical Ethics, 24(1), 11. 10.1186/s12910-023-00888-y 36793067 PMC9930063

[bibr6-15562646261428333] Canario Guzmán J. A. Orlich J. Mendizábal-Cabrera R. Ying A. Vergès C. Espinoza E. Soriano M. Cárcamo E. Beltrán B. Marrero E. R. M. Sepulveda R. Nieto Anderson C. de Colombi N. F. Lescano R. Pérez-Then E. Lang T. Alger J. (2022, December 23). Strengthening research ethics governance and regulatory oversight in Central America and the Dominican Republic in response to the COVID-19 pandemic: A qualitative study. Health Research Policy and Systems, 20(1), 138. 10.1186/s12961-022-00933-z 36564837 PMC9786418

[bibr7-15562646261428333] Clapp J. T. Gleason K. A. Joffe S. (2017, December). Justification and authority in institutional review board decision letters. Social Science & Medicine, 194(December 2017), 25–33. 10.1016/j.socscimed.2017.10.013 29059597

[bibr8-15562646261428333] Clarke D. L. (2014). Auditing the process of ethics approval for master's degrees at a South African university. South African Journal of Bioethics and Law, 7(1), 23–25. 10.7196/SAJBL.301

[bibr9-15562646261428333] Cleaton-Jones P. Grossman E. S. (2015). Things may not be as expected: Surprising findings when updating work load at the Wits Human Research Ethics Committee (Medical). South African Journal of Bioethics and Law, 8(1), 14–21. 10.7196/sajbl.339

[bibr10-15562646261428333] Coleman C. H. Khadem A. Reeder J. C. Sillo H. B. Gaspar R. Reis A. (2025, June 1). A world health organization tool for assessing research ethics oversight systems. Bulletin of the World Health Organization, 103(6), 403–409. 10.2471/BLT.24.292219 40511399 PMC12161159

[bibr11-15562646261428333] Council for International Organizations of Medical Sciences. (2016). International Ethical Guidelines for Health-related Research Involving Humans: Prepared by the Council for International Organizations of Medical Sciences (CIOMS) in collaboration with the World Health Organization (WHO) [Internet] (4th Ed.). Council for International Organizations of Medical Sciences. 10.56759/rgxl7405 40523065

[bibr12-15562646261428333] Decullier E. Lhéritier V. Chapuis F. (2005, October 17). The activity of French research ethics committees and characteristics of biomedical research protocols involving humans: A retrospective cohort study. BMC Medical Ethics, 6(E9), 1–10. 10.1186/1472-6939-6-9 PMC1323331 16229743

[bibr13-15562646261428333] Dixon-Woods M. Foy C. Hayden C. Al-Shahi Salman R. Tebbutt S. Schroter S. (2016, August 31). Can an ethics officer role reduce delays in research ethics approval? A mixed-method evaluation of an improvement project. BMJ Open, 6(8), e011973. 10.1136/bmjopen-2016-011973 PMC5013460 27580832

[bibr14-15562646261428333] Embrett M. Carson A. Sim M. Conway A. Moore E. Hancock K. Bielska I. (2025, March 26). Building resilient and responsive health research systems: Responses and the lessons learned from the COVID-19 pandemic. Health Research Policy and Systems, 23(1), 38. 10.1186/s12961-024-01229-0 40140981 PMC11938576

[bibr15-15562646261428333] Emmerich N. (2009). On the ethics committee: The expert member, the lay member and the absentee ethicist. Research Ethics, 5(1), 9–13. 10.1177/174701610900500103

[bibr16-15562646261428333] Eurasian Economic Commission. (2016). Annex to Decision of the Eurasian Economic Commission's Council “Amendments to the Rules of Good Clinical Practice of the Eurasian Economic Union”. EEC Council EAEU. Retrieved November 20, 2025, from https://eec.eaeunion.org/upload/medialibrary/632/Amendments-to-the-Rules-of-Good-Clinical-Practice-of-the-Eurasian-Economic-Union.pdf

[bibr17-15562646261428333] Fakhradiyev L. Shamsutdinova A. Kulkayeva G. Sarymsakova B. Menlayakova D. Manatova A. Saussakova S. Ibrayeva A. Tanabayeva S. Munir K. (2024). The functioning of ethics committees in Kazakhstan: Results and recommendations. Materia Socio Medica, 36(3), 192–198. 10.5455/msm.2024.36.192-198 39749153 PMC11693122

[bibr18-15562646261428333] Famenka A. (2011, April). Ethical review of biomedical research in Belarus: Current status, problems and perspectives. Revista Romana de Bioetica, 9(2), 74–83. Retrieved August 7, 2025, from https://pmc.ncbi.nlm.nih.gov/articles/PMC3372927/22701397 PMC3372927

[bibr19-15562646261428333] Famenka A. (2016, December). Research ethics in the context of transition: Gaps in policies and programs on the protection of research participants in the selected countries of central and Eastern Europe. Science and Engineering Ethics, 22(6), 1689–1706. 10.1007/s11948-015-9723-4 26548313 PMC4861682

[bibr20-15562646261428333] Ganesh D. Kalikar M. V. (2025, April–June). Role of lay person in ethics committee: Bridging expertise and public trust. Perspectives in Clinical Research, 16(2), 99–101. 10.4103/picr.picr_232_24 40322471 PMC12048089

[bibr21-15562646261428333] Green F. A. (2007). Further thoughts on the recruitment of REC lay members. Research Ethics, 3(1), 8–12. 10.1177/174701610700300104

[bibr22-15562646261428333] Greene S. M. Geiger A. M. (2006, August). A review finds that multicenter studies face substantial challenges but strategies exist to achieve institutional review board approval. Journal of Clinical Epidemiology, 59(8), 784–790. 10.1016/j.jclinepi.2005.11.018 16828670

[bibr23-15562646261428333] Happo S. M. Halkoaho A. Lehto S. M. Keränen T. (2016). The effect of study type on research ethics committees’ queries in medical studies. Research Ethics, 13(3-4), 115–127. 10.1177/1747016116656912

[bibr24-15562646261428333] Humphreys S. Thomas H. Martin R. (2014). Medical dominance within research ethics committees. Accountability in Research, 21(6), 366–388. 10.1080/08989621.2014.891944 24785996

[bibr25-15562646261428333] Hunt M. Tansey C. M. Anderson J. Boulanger R. F. Eckenwiler L. Pringle J. Schwartz L. (2016, June 21). The challenge of timely, responsive and rigorous ethics review of disaster research: Views of research ethics committee members. PLoS One, 11(6), e0157142. 10.1371/journal.pone.0157142 PMC4915681 27327165

[bibr26-15562646261428333] International Council for Harmonisation of Technical Requirements for Pharmaceuticals for Human Use. (2025). ICH Harmonised Guideline: Guideline for good clinical practice E6(R3). ICH Geneva. [online]. Retrieved August 7, 2025, from https://database.ich.org/sites/default/files/ICH_E6%28R3%29_Step4_FinalGuideline_2025_0106.pdf

[bibr27-15562646261428333] Kadam A. V. Patil S. Sane S. Shahabuddin S. M. Panda S. (2022, May–June). Challenges faced by ethics committee members in India during COVID-19 pandemic: A mixed-methods exploration. Indian Journal of Medical Research, 155(5&6), 461–471. 10.4103/ijmr.ijmr_1095_22 36348593 PMC9807194

[bibr28-15562646261428333] Kebenei E. K. Cheruiyot D. Msee G. C. Nguya J. Kiplagat T. K. Bukusi E. A. (2024, April–June). The impact of COVID-19 pandemic on the research portfolio and approval turnaround time at the Kenya medical research institute. Indian Journal of Medical Ethics, 9(2), 109–114. 10.20529/IJME.2024.013 38755766

[bibr29-15562646261428333] Khatri R. B. Endalamaw A. Erku D. Wolka E. Nigatu F. Zewdie A. Assefa Y. (2023, November 30). Preparedness, impacts, and responses of public health emergencies towards health security: Qualitative synthesis of evidence. Archives of Public Health, 81(1), 208. 10.1186/s13690-023-01223-y 38037151 PMC10687930

[bibr30-15562646261428333] Kornioti N. Seedall C. Kalaitzaki K. Marcou A. (2024). Recommendations for expediting ethics review during global crisis, a report for PREPARED. PREPARED, Pyla, Cyprus. [online]. Retrieved August 7, 2025, from https://prepared-project.eu/

[bibr31-15562646261428333] Kudaibergenova T. Ibrahim M. Jain N. Vetra J. (2023, July). Documentary assessment of the abilities of Kyrgyzstan's research ethics committees during public health emergency and non-emergency situations. Journal of Empirical Research on Human Research Ethics, 18(3), 99–108. 10.1177/15562646231176711 37203158 PMC10251458

[bibr32-15562646261428333] Legood G. (2005). The recruitment and role of lay members. Research Ethics, 1(4), 135–138. 10.1177/174701610500100407

[bibr33-15562646261428333] McGahan E. Berkman J. Milne D. Terrill B. Gear R. Gardiner S. Eckstein L. Nicol D. Taylor N. Winship I. McWhirter R. Nisselle A. Lodge J. M. McInerney-Leo A. M. (2025, July). Empowering human research ethics committees to review genomics applications: Evaluating the utility of a custom online education resource. European Journal of Human Genetics, 33(7), 945–955. 10.1038/s41431-025-01846-5 40247136 PMC12229321

[bibr34-15562646261428333] Mirpuri E. García-Álvarez L. Acín-Gericó M. T. Bartolomé B. Delgado Bolton R. C. San-Martín M. Vivanco L. (2022, June 16). Characterization of factors predicting a favorable opinion of research applications submitted for an ethical review process. Frontiers in Medicine, 9, 878786. 10.3389/fmed.2022.878786 35783641 PMC9243650

[bibr35-15562646261428333] Moodley K. Kabanda S. M. Kleinsmidt A. Obasa A. E. (2021, September 25). COVID-19 underscores the important role of clinical ethics committees in Africa. BMC Medical Ethics, 22(1), 131. 10.1186/s12910-021-00696-2 34563181 PMC8465788

[bibr36-15562646261428333] Mrisho M. Essack Z. (2021, December). Understanding constraints and enablers of turnaround time for ethics review: The case of institutional review boards in Tanzania. Journal of Empirical Research on Human Research Ethics, 16(5), 514–524. 10.1177/15562646211026855 34180729 PMC8530844

[bibr37-15562646261428333] National Statistical Center of the Kyrgyz Republic. (2024). Женщины и мужчины Кыргызской Республики: 2019–2023. Бишкек: 2024–307 с. ISBN 978–9967–28–558–3. Retrieved August 7, 2025, from https://www.stat.gov.kg/media/publicationarchive/31330217-aae2-48b8-acef23e847e3c7ca.pdf

[bibr38-15562646261428333] Nicholls S. G. Taylor H. A. James R. Anderson E. E. Friesen P. Schonfeld T. Summers E. I. (2023). A cross sectional survey of recruitment practices, supports, and perceived roles for unaffiliated and non-scientist members of IRBs. AJOB Empirical Bioethics, 14(3), 174–184. 10.1080/23294515.2023.2180107 36821084 PMC10444906

[bibr39-15562646261428333] Rani M. Chawla N. Wadhwa N. Mathur R. Jinks T. Das P. Rijal S. (2024, September 20). A transformative solution to build effective, transparent, and resilient "fit-for-purpose" national health research ethics systems. Health Research Policy and Systems, 22(1), 131. 10.1186/s12961-024-01219-2 39304929 PMC11414025

[bibr40-15562646261428333] Salamanca-Buentello F. Katz R. Silva D. S. Upshur R. E. G. Smith M. J. (2024, April 16). Research ethics review during the COVID-19 pandemic: An international study. PLoS One, 19(4), e0292512. 10.1371/journal.pone.0292512 PMC11020390 38626030

[bibr41-15562646261428333] Sarvmsakova B. (2009). Central Asia–Developing a capacity building approach to ethical review. Asian Bioethics Review, 1(2), 168–170. Retrieved August 7, 2025, from http://hdl.handle.net/20.500.12424/233356

[bibr42-15562646261428333] Semenova Y. Lim L. Salpynov Z. Gaipov A. Jakovljevic M. (2024, April 15). Historical evolution of healthcare systems of post-soviet Russia, Belarus, Kazakhstan, Kyrgyzstan, Tajikistan, Turkmenistan, Uzbekistan, Armenia, and Azerbaijan: A scoping review. Heliyon, 10(8), e29550. 10.1016/j.heliyon.2024.e29550 PMC11036062 38655295

[bibr43-15562646261428333] Sidaway M. Collett C. Kolstoe S. E. (2023, July 3). Evidence from UK research ethics committee members on what makes a good research ethics review, and what can be improved. PLoS One, 18(7), e0288083. 10.1371/journal.pone.0288083 PMC10317218 37399215

[bibr44-15562646261428333] Silverman H. Sleem H. Moodley K. Kumar N. Naidoo S. Subramanian T. Jaafar R. Moni M. (2015, April). Results of a self-assessment tool to assess the operational characteristics of research ethics committees in low- and middle-income countries. Journal of Medical Ethics, 41(4), 332–337. 10.1136/medethics-2013-101587 24748650

[bibr45-15562646261428333] Solomon S. (2016). Too many rationales, not enough reason: A call to examine the goals of including lay members on institutional review boards. Accountability in Research, 23(1), 4–22. 10.1080/08989621.2014.956865 26528638

[bibr46-15562646261428333] Strosberg M. A. Gefenas E. Loue S. Philpott S. (2013, December). Building research ethics capacity in post-communist countries: Experience of two fogarty training programs. Journal of Empirical Research on Human Research Ethics, 8(5), 28–39. 10.1525/jer.2013.8.5.28 24384514 PMC4259573

[bibr47-15562646261428333] Supreme Council of the Kyrgyz Republic. (2025). Кодекс КР от 23 января 2025 года № 23 “Трудовой кодекс Кыргызской Республики”. Retrieved August 7, 2025, from https://cbd.minjust.gov.kg/3-45/edition/25298/ru

[bibr48-15562646261428333] Tattoli L. Abenante B. Cavallo Perin P. Maddalena M. Lupariello F. (2025, May 26). Ethical issues and the role of the ethics committees during COVID-19 research in pandemic era: A focus on an Italian ethics committee. Frontiers in Public Health, 13, 1537863. 10.3389/fpubh.2025.1537863 40491996 PMC12146303

[bibr49-15562646261428333] Taur S. R. Bavdekar S. B. Thatte U. M. (2011, October–December). Survey of ethics committee protocol approval letters: Compliance with schedule Y/ICMR guidelines 2006. Indian Journal of Medical Ethics, 8(4), 214–216. 10.20529/IJME.2011.083 22106659

[bibr50-15562646261428333] Tumilty E. Young J. James R. Serpico K. Johnson A. Anderson E. E. (2025, December). A scoping review of empirical research on research ethics board membership and expertise. Journal of Empirical Research on Human Research Ethics, 20(5), 275–285. 10.1177/15562646251376747 41032647 PMC12490803

[bibr51-15562646261428333] World Health Organization. (2000). Operational guidelines for ethics committees that review biomedical research - TDR/PRD/ETHICS/2000.1. WHO Geneva. Retrieved August 7, 2025, from https://iris.who.int/handle/10665/66429

[bibr52-15562646261428333] World Health Organization. (2011). Standards and operational guidance for ethics review of health-related research with human participants. Health Ethics & Governance Team. World Health Organization (WHO) Geneva. Retrieved August 7, 2025, from https://www.who.int/publications/i/item/9789241502948

[bibr53-15562646261428333] World Health Organization. (2023). WHO tool for benchmarking ethics oversight of health-related research involving human participants. Health Ethics & Governance Team. World Health Organization (WHO) Geneva. Retrieved August 7, 2025, from https://www.who.int/publications/i/item/9789240076426

[bibr54-15562646261428333] World Medical Association. (2025, January 7). World medical association declaration of Helsinki: Ethical principles for medical research involving human participants. JAMA, 333(1), 71–74. 10.1001/jama.2024.21972 39425955

[bibr55-15562646261428333] Yuan M. Lin H. Wu H. Yu M. Tu J. Lü Y. (2021, January 12). Community engagement in public health: A bibliometric mapping of global research. Archives of Public Health, 79(1), 6. 10.1186/s13690-021-00525-3 33436063 PMC7801880

